# Prevalence of dance-related injuries and associated risk factors among children and young Chinese dance practitioners

**DOI:** 10.1097/MD.0000000000036052

**Published:** 2023-11-24

**Authors:** Ryan K.H. Hung, Patrick S.H. Yung, Samuel K.K. Ling, Dino Samartzis, Cliffton Chan, Claire Hiller, Esther T.C. Cheung, Veronika Schoeb, Brenton Surgenor, Arnold Y.L. Wong

**Affiliations:** a Department of Orthopaedics and Traumatology, The Chinese University of Hong Kong, Hong Kong, SAR, China; b Department of Orthopedic Surgery, Rush University Medical Center, Chicago; c Department of Health Sciences, Macquarie University, New South Wales, Australia; d Faculty of Medicine and Health, The University of Sydney, New South Wales, Australia; e Department of Rehabilitation Sciences, The Hong Kong Polytechnic University, Hong Kong SAR, China; f School of Health Sciences (HESAV), University of Applied Sciences and Arts Western Switzerland (HES-SO), Lausanne, Switzerland; g Victorian College of the Arts, The University of Melbourne, Victoria, Australia.

**Keywords:** Chinese dance, dance injury, epidemiology, injury rate, musculoskeletal pain, risk factors

## Abstract

While Chinese dance is a popular dance genre among Chinese teenagers and adults, little is known regarding the prevalence of dance-related injuries or factors associated with such injuries among Chinese dance practitioners. The current cross-sectional study aimed to determine the prevalence of dance-related injuries and their associated risk factors among young Chinese dance practitioners in Hong Kong. Online surveys were distributed to dancers through local dance associations, while paper-based surveys were distributed to young Chinese dance performers during the 54^th^ School Dance Festival in Hong Kong. Self-reported hours of dancing, injuries in the last 12 months, injury sites, and related factors were collected. The injury rate, 12-month prevalence of dance-related injuries were determined. Risk factors for common dance injuries were analyzed using separate multivariate regression models. A total of 175 children (aged 10–14 years) and 118 young (aged 15–24 years) Chinese dance practitioners provided their dance injury information. Young dancers had a significantly higher injury rate (6.5 injuries vs 4.6 injuries/1000 dance hours) and 12-month prevalence (52.5% vs 19.4%) than their child counterparts. The most commonly injured sites were the knee (children:7.4%; young:15.3%), lower back (children: 4.6%; young: 9.5%), and ankles (children: 5.1%; young: 16.9%). Age was a significant independent risk factor for dance-related injuries to the upper back, lower back, and pelvis/buttock (odds ratios ranging from 1.2 to 1.3/additional years). Additionally, height was a significant independent risk factor for lower limb injury (odds ratios ranging from 1.0–1.1/additional centimeter). Collectively, young Chinese dance practitioners are more vulnerable to dance-related injuries than are child dancers. Older age increases the risk of trunk injuries, whereas taller dancers are more likely to sustain lower-limb injuries. Future research should determine the mechanisms underlying dance-related injuries among these dancers.

## 1. Introduction

Chinese classical dance and Chinese folk dance are the most popular dance genres among teenagers in Hong Kong and China given its unique cultural elements. Developed in the twentieth century, it is a form of modern concert dance that incorporates acrobatics, Chinese martial arts, Chinese opera, indigenous performance elements from various Chinese ethnic groups, and western ballet.^[1,2]^ It also included some Chinese customs, traditional aesthetics, and etiquettes. While dancing improves aerobic capacity, increases muscle strength and endurance, improves flexibility and balance, lowers body fat composition,^[3]^ many amateur, preprofessional, or professional dancers sustain dance-related injuries.^[4–7]^

A systematic review reported that the prevalence of lumbosacral pain, patellofemoral pain, and snapping hip in ballet dancers were 62%, 29%, and 91%, respectively.^[8]^ Another critical review^[5]^ found that the prevalence of back injuries ranged from 7%^[9]^ to 62%^[10]^ regardless of age or dance genres. Likewise, the reported 12-month prevalence of dance-related injuries among pre-professional or professional ballet dancers ranged from 17%^[6]^ to 67.4%,^[7]^ which was comparable to other sports (e.g., soccer, handball, or runners).^[11–16]^ However, because most of the prior studies mainly evaluated dance-related injuries of ballet, modern, contemporary or break dance in western countries,^[8,17–19]^ there is a paucity of research that evaluated the prevalence of dance injures and related risk factors among Chinese dance practitioners. Although Dang and coworkers revealed that pre-professional or professional Chinese folk dance dancers had lower mean injury rate (3.4 injuries/dancers) than contemporary dancers (4.9 injuries/dancers),^[1]^ their findings cannot be generalized to amateur dancers, who comprise the majority of Chinese dance partitioners.

Although lower extremity dance injuries are common among dancers at different skill levels,^[4,19,20]^ different dance genres may have their unique dance injury pattens and risk factors.^[4,19]^ For instance, ballet dancers frequently sustain foot and ankle injuries, given their unique dance techniques (e.g., demi-pointe and pointe).^[8]^ Conversely, upper limb injuries are relatively common among hip hop dancers (especially breakers) because of their frequent upper limb weight-bearing manoeurves.^[18]^ Chinese dance usually requires dancers to jump, leap, turn, tumble using a single-foot take-off and landing in multiple directions. It is conceivable that Chinese dance practitioners are more likely to have ankle and foot injuries. Further, because Chinese dance performances often involve back arching and leg splitting, these body parts may be at risk of acute or overuse injuries. However, no previous large-scale study has investigated the prevalence, injury patterns, or factors associated with the increased prevalence of dance injuries among young Chinese dance practitioners, which can help inform the development of specific strategies to prevent or manage Chinese dance-related injuries.

Given the above, the current study aimed to investigate the prevalence, injury patterns, and associated risk factors of dance injuries among children and youth dancers practicing Chinese dancing in Hong Kong.

## 2. Methods

### 2.1. Study design

This study was a subgroup analysis of a large-scale cross-sectional epidemiological project investigating the prevalence rates and characteristics of dance injuries, as well as associated risk factors, among amateurs and professional dancers in Hong Kong.^[4]^ The study was approved by the Human Subjects Ethics Subcommittee of The Hong Kong Polytechnic University (HSEARS 20180130006). This study was reported according to the Strengthening the Reporting of Observational Studies in Epidemiology (STROBE) checklist,^[21]^ and the Checklist for Reporting Results of Internet E-Surveys (CHERRES) guidelines.^[22]^

### 2.2. Participants

There were 2 main target groups recruited by convenience sampling. The first target group included primary and secondary school students who participated in the 2019 School Dance Festival competition in Hong Kong (approximately 1500 dance practitioners). Data from this group was collected using online and paper-based questionnaires. The second target group was the general public, who regularly practiced Chinese dance in the community but not in primary/secondary schools. Data from this group was collected using an identical online questionnaire promoted on the Hong Kong Dance Alliance Limited website where dancing enthusiast will visit (approximately 3000 members). Parental consent was unnecessary for children under 18 years of age to participate in the current survey because the collected data were anonymous and considered low risk. Optionally, participants could leave their email addresses in the questionnaire to receive a summary of their research findings. By assuming a 29% response rate as reported in a previous large-scale dance survey study,^[6]^ it was anticipated to receive 1305 completed questionnaires.

Data on recreational dancers who practiced only Chinese dance were selected for the current analysis to minimize the confounding effects of other dance genres. Chinese dance practitioners were divided into child (10–14 years old) and youth groups (15–24 years old) groups based on age. The stratification of age groups for analysis was based on 2 reasons: (i) the classification from the World Health Organization (WHO), which defined “youth” as individuals aged between 15 and 24 years,^[23]^ and (ii) the fact that dancers who attained a higher professional level had greater injury risks due to cumulative training effects, advanced training levels (difficulty), and/or prior history of dance injuries in more experienced dancers.^[19]^ This stratification method allowed for the investigation of age- and skill-specific injury patterns.

### 2.3. Procedures

The data were collected between December 2018 and May 2019. The survey was promoted in several phases: promotional posters for the online survey were mailed to the 659 schools that participated in the Schools Dance Festival by the Schools Dance Association in December 2018. The responsible dance teachers were encouraged to display the posters in their schools; promotional posters for the online survey were manually distributed to 50 dance teachers who attended the briefing session of the School Dance Festival in April 2019; and a team of 6 research personnel promoted the paper-based questionnaire to primary and secondary school dance teachers during the 5-day Schools Dance Festival winner performance in May 2019. The teachers introduced research personnel who explained the purpose of the questionnaire to potential respondents. Students were informed that their participation in the survey was voluntary without any incentive. If the students required clarification of any items in the paper-based questionnaire during the 5-day Schools Dance Festival winner performance, our researchers provided immediate assistance. The public was invited to complete the online survey voluntarily through a series of advertisements published on the Hong Kong Dance Alliance Limited website from February to May 2019.

All paper-based questionnaires were stored in a locked cabinet in a locked room, while online data was downloaded as an Excel file and stored in an encrypted computer by an authorized researcher. The authorized researcher manually entered the data written in the paper-based questionnaires into an Excel file. All data was de-identified to ensure confidentiality.^[24]^

### 2.4. Online/paper-based questionnaire

The self-administered online/paper-based questionnaire was modified from similar questionnaires,^[20,25,26]^ and was developed by a panel of 4 physiotherapists, an orthopedic surgeon specializing in sports medicine, 2 dance scientists, a sports psychologist, and an epidemiologist, with an average of 17 years of experience in clinical practice and/or musculoskeletal research. The online survey was created using an online tool (https://typeform.com). The first page of the online/paper-based questionnaire provided the details of the study. The remaining questionnaire comprised 4 sections: participants’ demographics (e.g., sex, age, height, weight, and comorbidities such as scoliosis); dance history (e.g., dance genres practiced, average weekly practice hours, and years of regular dance practice); history of dance-related injury (e.g., number of dance-related injuries in the last 12 months and the resulting injured body parts); and injury-related management and psychology (Supplemental file, http://links.lww.com/MD/K781). In this survey, dance-related injury was defined as any physical (e.g., numbness, weakness, pain, or other physical symptoms) or psychological condition (e.g., fear, distress) following an injury that prohibited a person from fully participating in scheduled dance activities (practice or performance) that would have happened otherwise. Some items had a “not applicable” option. The online questionnaire had some mandatory items. Respondents could use the “back” button to amend the responses before submission. The online and paper-based questionnaires had 69 items, and the estimated completion time was 20 minutes. However, the online questionnaire used an adaptive questioning approach to skip irrelevant items based on respondents’ responses of previous items. The online and paper-based questionnaires were piloted on 10 and 20 teenage dancers (aged 10 to 18 years), respectively, to ascertain the comprehensibility of the questions. The questions were modified according to the feedback. Their data was not included in the data analysis.

### 2.5. Data analysis

All statistical analyses were conducted using SPSS version 25.0 (IBM, Armonk, NY, USA), with the significance level set at *P* < .05. Incomplete questionnaires were excluded from data analysis. Online questionnaire submitted within 5 minutes or after 40 minutes were also excluded from analysis. Descriptive analyses of demographic data (e.g., age, sex, height, weight, and body mass index), training characteristics (e.g., average weekly practicing hours and years of regular dance practice), and history of dance injury (e.g., body regions injured in the last 12 months) among children and young Chinese dance practitioners have been reported. The injury rate was calculated by dividing the number of injuries by 1000 dancing hours of exposure (practice and performance). Mann–Whitney U tests were used to compare the demographic data between the 2 groups. The injury rate and 12-month prevalence of dance injuries in various body parts have been reported previously. Chi-square tests were used to compare differences in prevalence and injury rates. Odds ratios (ORs) were calculated to evaluate the factors associated with increased odds of sustaining dance-related injuries in different body regions. Separate stepwise multivariate logistic regression models were used to identify factors associated with commonly injured sites (i.e., the trunk and lower limbs) in the 2 groups. Specifically, demographic or dance-related factors (e.g., diagnosed scoliosis and average weekly practicing hours) that showed significant differences between dancers with and without injuries were entered as independent variables, whereas each selected body region was the dependent variable.^[4]^ The resulting ORs and 95% confidence intervals were reported. The adopted dance injury prevention and management methods were reported to help understand the dance injury management strategies of these young dancers (regardless of their age group). Differences in dance management strategies between dancers with and without a history of dance injuries were compared using the chi-square test.

## 3. Results

One thousand 4 hundred and sixty-five respondents completed the online and paper-based questionnaires. The response rate could not be calculated because the total number of people who saw promotional posters in schools and on the website was unknown, but overall responses were better than expected. Although there were no limitations on the eligibility of the dancers’ performance levels, no self-proclaimed preprofessional or professional Chinese dance practitioners completed the online questionnaire. In the student group, 180 online questionnaires and 658 paper-based questionnaires were collected. In total, 627 online questionnaires were collected from a public group. The completion rate was 30.8%. Of all collected questionnaires, 308 (183 children and 125 youth) dancers practiced Chinese dance alone. However, 15 participants had missing data from their questionnaires. Therefore, data from 293 children (175 children and 118 youth) were used for data analysis.

The demographic characteristics of the respondents are presented in Table 1. The average age of the youth group was significantly higher than that of the child group. For the youth group aged, they were significantly heavier and taller than the child group. More than twice the number of youth dancers had self-reported scoliosis compared to child dancers. The youth group had a significantly higher average weekly practice duration and significantly more years of regular dance practice and performance than the child group. In short, youth dancers had a greater existing and cumulative training load than child dancers did.

**Table 1:** Demographic data of the respondents.

	Overall	Children (10–14 yr)	Youth (15–24 yr)
	(n = 293)	(n = 175)	(n = 118)
Age (yr)	14.4 ± 3.7	11.9 ± 1.4*	18.1 ± 2.7*
Gender (female)	90.4% (n = 265)	88.6% (n = 155)	93.2% (n = 110)
Weight (kg)	45.3 ± 9.6	41.0 ± 9.1*	51.0 ± 7.0*
Height (cm)	156.0 ± 9.6	151.6 ± 9.3*	162.0 ± 5.9*
Body mass index	18.4 ± 2.7	17.6 ± 2.8*	19.5 ± 2.2*
Scoliosis	16.4% (n = 48)	11.4%* (n = 20)	23.7%* (n = 28)
Average weekly practice hours	6.7 ± 10.3	4.9 ± 8.2*	9.3 ± 12.4*
Years of regular dance practice	7.3 ± 6.1	5.6 ± 6.4*	9.7 ± 4.7*
Years of regular performance	6.0 ± 3.7	4.5 ± 2.7*	8.0 ± 4.0*
Number of competitions per year	3.9 ± 3.8	3.6 ± 2.9	4.2 ± 4.9
Number of performances per year	5.2 ± 4.5	4.7 ± 4.4*	6.0 ± 4.6*
Self-perceived anxiety	9.6% (n = 28)	6.9% (n = 12)	13.6% (n = 16)
Self-perceived depressive symptoms	7.8% (n = 23)	6.3% (n = 11)	10.2% (n = 12)

* *P* < .05; descriptive statistics represented as percentage and (sample size) and mean ± standard deviation where applicable.

The injury rates of children were 1.9 injuries/1000 hours of dance exposure less than those of youth dancers, reaching statistical significance. The 12-month prevalence and ORs of Chinese-dance injuries in different body regions of the 2 groups are displayed in Tables 2 and 3, respectively. Young dancers (52.5%) demonstrated a significantly higher overall 12-month prevalence of dance injuries than child dancers (19.4%), regardless of the body region. Youth dancers also had a significantly higher 12-month prevalence of multisite injuries than did child dancers. The 3 most commonly injured sites in the last 12 months among Chinese dance practitioners were the knee (10.6%), lower back (10.6%), and ankle (9.9%). The youth group demonstrated significantly higher 12-month prevalence rates of injuries in the neck, upper back, lower back, pelvis/buttock, ankle, and feet/toe regions than did the child group. Similarly, youth dancers had significantly higher risks of injury at the upper back, lower back, pelvis/buttock, ankle, and feet/toe, as well as multisite injuries in the last 12 months than their child counterparts (ORs ranging from 3.5 to 13.8). Interestingly, although the knee was the most common Chinese dance-related injury site, there was no statistically significant difference in the 12-month prevalence of knee injuries between the 2 groups.

**Table 2:** Injury rate and prevalence rates of dance-related injuries at different body parts.

	Overall (n = 293)	Children (10–14 yr) (n = 175)	Youth (15–24 yr) (n = 118)
Dance injury rate in Chinese dance practitioners			
Number of injuries per 1000 dancing hours	5.4	4.6*	6.5*
12-month prevalence rates at different body parts			
At least 1 injured site	32.7% (n = 96)	19.4%* (n = 34)	52.5%* (n = 62)
More than 1 injured site	16.7%*(n = 49)	8.6%* (n = 15)	28.8%* (n = 34)
Head	0.7% (n = 2)	1.1% (n = 2)	0% (n = 0)
Neck	3.1% (n = 9)	1.1%* (n = 2)	6.0%* (n = 7)
Shoulder	2.7% (n = 8)	1.1% (n = 2)	5.1% (n = 6)
Upper arm	0.3% (n = 1)	0.6% (n = 1)	0% (n = 0)
Elbow	0.7% (n = 2)	1.1% (n = 2)	0% (n = 0)
Forearm	0.7% (n = 2)	0.6% (n = 1)	0.8% (n = 1)
Wrist/finger	2.4% (n = 7)	1.7% (n = 3)	3.4% (n = 4)
Chest/ribs	1.0% (n = 3)	1.1% (n = 2)	0.8% (n = 1)
Abdomen	0.7% (n = 2)	1.1% (n = 2)	0% (n = 0)
Upper back	5.1% (n = 15)	2.3%* (n = 4)	9.3%* (n = 11)
Lower back	10.6% (n = 31)	4.6%* (n = 8)	19.5%* (n = 23)
Pelvis/buttock	3.8% (n = 11)	0.6%* (n = 1)	8.5%* (n = 10)
Thigh	6.1% (n = 18)	4.6% (n = 8)	8.5% (n = 10)
Knee	10.6% (n = 31)	7.4% (n = 13)	15.3% (n = 18)
Lower leg	4.1% (n = 12)	4.6% (n = 8)	3.4% (n = 4)
Ankle	9.9% (n = 29)	5.1%* (n = 9)	16.9%* (n = 20)
Feet/toe	5.8% (n = 17)	1.1%* (n = 2)	12.7%* (n = 15)

* *P* < .05; the injury rate is presented as mean, while prevalence rate represented as percentage and (sample size).

**Table 3:** Odds ratios for Chinese dance-related injury at different body parts for the youth dancer group with reference to the child dancer group.

Body region	Odds ratio (95% confidence interval)	*P* value
At least 1 body part	4.6 (2.7–7.8)	<.001
Multiple regions	4.0 (2.0–8.1)	<.001
Head	NS	NS
Neck	NS	NS
Shoulder	NS	NS
Upper arm	NS	NS
Elbow	NS	NS
Forearm	NS	NS
Wrist/finger	NS	NS
Chest/ribs	NS	NS
Abdomen	NS	NS
Upper back	3.7 (1.1–12.1)	.029
Lower back	4.4 (1.9–10.5)	.001
Pelvis/buttock	13.8 (1.7–109.6)	.013
Thigh	NS	NS
Knee	NS	NS
Lower leg	NS	NS
Ankle	3.5 (1.4–7.5)	.006
Feet/toe	10.9 (2.4–49.1)	.002

NS = not significant.

The results of the multivariate logistic regression models are presented in Table 4. Age was a significant factor associated with Chinese dance-related injury in the upper back, lower back, and pelvis/buttock (ORs ranging from 1.2 to 1.3) after considering factors (i.e., height, weight, self-reported scoliosis, and average weekly practicing hours) that showed significant differences between dancers with and without injuries. Interestingly, height was the only significant independent risk factor for Chinese dance-related injuries in the knee, ankle, and toe/feet regions after considering age, weight, scoliosis, and average weekly practice hours.

**Table 4:** Multivariate analyses of risk factors for predicting Chinese dance-related injury at different body regions in the last 12 months.

Injury site	Age (odds ratio, 95% confidence interval)	Height (odds ratio, 95% confidence interval)
Upper back	1.2 (1.0–1.4); *P* = .013	---
Lower back	1.2 (1.1–1.4); *P* < .001	---
Pelvic/buttock	1.3 (1.1–1.5); *P* = .006	---
Knee	---	1.0 (1.0–1.1); *P* = .031
Ankle	---	1.1 (1.0–1.1); *P* = .014
Feet/toe	---	1.1 (1.0–1.2); *P* = .017

Age, height, weight, diagnosed scoliosis and average weekly practicing hours were entered in these models.

More than half of the participants with a previous dance injury chose to ignore the injury and continued dancing (Table 5). The 3 most adopted approaches for managing dance-related injuries are rest, stretching, and ice therapy. The 3 most prevalent strategies adopted by dancers to prevent dance injuries are warm-up, regular breaks, and cool-down. There were no significant differences in the frequency of various preventive or management strategies adopted by dancers with and without a history of dance injuries.

**Table 5:** Choices in the management and prevention of dance injury among Chinese dance practitioners.

Selected choices	With dance injury history (n = 96)	Without dance injury history (n = 194)	*P* value
Ignoring the injury and continue to dance	52.1 % (n = 50)	NNA	ANA
Managing the injury by resting	62.5% (n = 60)	ANA	ANA
Managing the injury by stretching	30.2% (n = 29)	ANA	ANA
Managing the injury by ice therapy	28.1% (n = 27)	ANA	ANA
Preventing the injury by doing warm up	68.8% (n = 66)	72.2% (n = 140)	NS
Preventing the injury by taking regular breaks	53.1% (n = 51)	53.1% (n = 103)	NS
Preventing the injury by doing cool down	46.9% (n = 45)	49.0% (n = 95)	NS

NA = not applicable, NS = not significant.

## 4. Discussion

This is the first study to investigate the injury rate, 12-month prevalence, patterns of dance-related injuries, and associated risk factors among young amateur dancers practicing Chinese dancing. The estimated injury rates of these young dancers ranged from 4.6 to 6.5 injuries/1000 dance hours. Age and height were the 2 factors associated with injuries in the different body regions.

The injury rate found in the current study was higher than that of young recreational dancers practicing ballet, contemporary dance, or breakdance (ranging from 3.1–4.5 injuries/1000 dance hours).^[27–29]^ This indicates that the injury risk associated with practicing Chinese dance may be higher than those practicing other dance genres. Importantly, since our respondents were relatively younger than those in the previous literature, the higher injury rate of young Chinese dance practitioners is alarming. Given that prior injury is a known risk factor for reinjury in various dance disciplines,^[30]^ the high prevalence of dance injuries among young amateur Chinese dance practitioners highlights the importance of developing effective prevention and rehabilitation strategies for dance injuries among these dancers.

The most commonly injured sites among young Chinese dance practitioners were the knee, lower back, and ankle. This injury pattern is comparable to modern dance.^[6]^ Similarly, the knee and ankle were found to be commonly injured in ballet.^[8]^ These lower limb injuries may be related to repeated jumping, spinning, tiptoe maneuvers in multiple planes, which result in overuse injuries such as hamstring strain, patellofemoral pain, and Achilles tendinopathy, and/or chronic ankle instability.^[2,8]^ Although the causes of injuries in young Chinese dance practitioners remain to be explored, Chinese dance moves may share some common characteristics with modern dance (e.g., changes of the movement speed) and ballet (e.g., demi-pointe) that heighten the risk of lower limb dance-related injuries in these dancers.

Age is an important factor for the prediction of injuries in trunk and pelvis. Compared to the child group, the youth group had significantly higher 12-month prevalence of dance injuries at multiple body parts. Our findings echo those of previous studies,^[19,31]^ highlighting age as a significant risk factor for dance injuries regardless of dance genres. Experienced dancers usually play more demanding roles in performance, leading to relatively higher physical, psychological, and artistic demands during dance training and performance.^[31]^ Therefore, the current study found that average weekly practice hours and number of dance performances per year in the youth group were nearly double those in the child group. As our respondents were relatively young, other physiological changes (e.g., growth spurt and menarche) might have confounded the results. Future studies should consider these factors in their analyses.

Height is a risk factor for Chinese dance-related injuries of the knee, ankle, and foot/toe. This finding concurs with previous literature,^[32]^ although contradictory findings have also been reported.^[33–35]^ Campoy et al^[32]^ found that classical ballet dancers who sustained dance injuries had a higher mean height, whereas tap/folk dancers with dance injuries had a lower average height. Although speculative, a tall ballet dancer (longer lever arm) might place a dancer foot and tissues in great demand because of mechanical disadvantages, resulting in an increased risk of injury.^[36]^ Conversely, a short tap/folk dancer (shorter lever arm) might need to exhaust more lower-limb muscle strength to create a loud rhythm tap than a tall tap/folk dancer. Chinese dance involves plenty of spinning or jumping using forefoot or toes for takeoff and landing, like ballet. Biomechanically, a taller dancer might create a longer lever arm and cause greater absolute torque and compression over the distal joint surfaces of the metatarsophalangeal region or the ankle than a shorter dancer does. Future biomechanical studies should investigate the underlying mechanisms between body height and dance injuries to the ankles and feet in Chinese dance practitioners.

The post-dance injury management strategies adopted by the current participants are worrisome. More than half of the participants with a history of injury ignored their injuries and continued to dance without medical or allied health management. Previous dance injuries are known to increase the risk of future dance injuries.^[37]^ Untreated injuries may lead to cumulative tissue damage or overuse, which further contributing to reinjuries. As Chinese dance is a whole-body sport, injury at one part of the kinetic chain may cause compensatory movement along the chain, leading to injuries/problems in other body regions.^[38]^ Therefore, timely rehabilitation, identification of post-injury abnormal biomechanics, and retraining of proper dance techniques are crucial to prevent dance-related reinjuries.

### 4.1. Clinical implications

The current study revealed that truncal and lower limb regions were commonly injured in young Chinese dancers. Dynamic spinal and lower-limb stabilization exercises should be included in routine training during Chinese dance practice. Spinal motor control exercises have been shown to effectively improve pain, postural endurance and resilience, which is crucial in dancing.^[39,40]^ In the lower limb, poor vertical jump height is negatively related to the total number of days off after dance injuries.^[41]^ Further, improved isokinetic muscle strength, adequate flexibility, and enhanced muscle balance can reduce dance injuries by 15% to 50%.^[42]^ Since some female dancers misunderstand that muscle strengthening may increase their muscle mass and affects their aesthetic contours (body shape),^[43]^ health education emphasizing on the benefits of strengthening and conditioning for improving dance performance and preventing dance-related injuries should be given to dancers.

While age and height are non-modifiable factors related to dance injuries among Chinese dance practitioners, other dance injury prevention strategies (e.g., knee and ankle proprioception training, breaks, warm-up, and cool-down) can be implemented. Future clinical trials should investigate the effectiveness of these strategies in lowering the risk of dance-related injuries among Chinese dance practitioners.

### 4.2. Limitations

Our study had several limitations. First, all data (e.g., height, hours of dancing, and injury) were self-reported, it was possible that the reported pain or injuries might not be dance-related^.[44]^ However, since a clear definition of dance-related injury was given in the questionnaire, the reported injuries should be dance-related. Nevertheless, detailed physical assessments and medical diagnoses of injuries should be included in future studies to better understand the mechanisms underlying Chinese dance-related injuries. Second, some potential physical risk factors (e.g., proprioceptive deficits, aerobic capacity, muscle strength, and flexibility) were not assessed and considered in multivariate analyses. Future studies should address this limitation by including relevant physical examinations of participants. Third, given the self-reported nature of the questionnaire, weight, height, and diagnosis of scoliosis could not be validated. However, this is a common limitation in similar epidemiological studies. Fourth, because most of our participants were young amateur Chinese dancers, their findings cannot be generalized to older or professional dancers. Future studies should recruit preprofessional and professional Chinese dancers to determine the prevalence of dance-related injuries and the associated risk factors. Fifth, the current questionnaire comprised 69 items, which might discourage people from completing it. Future studies could use a shorter questionnaire to improve the completion rate.

## 5. Conclusions

Dance injuries are common among young Chinese dance practitioners. The prevalence and pattern of dance injuries were comparable to those in other dance genres. The 3 most commonly injured sites were the knee, lower back, and ankle. Age and height were 2 significant independent factors associated with increased Chinese-dance injuries in certain body parts. Youth dancers are more vulnerable to dance injuries than their children counterparts. While the current study underscores that some individuals are at risk of sustaining injuries during Chinese dance practice, future mechanistic studies are warranted to reveal the mechanisms underlying this high-risk group, so that proper strategies can be developed to treat or prevent dance-related injuries in these dancers.

## Acknowledgments

The current project was funded by the Health Care and Promotion Fund (grant number: 01170248). The authors have no financial or other conflicts of interest to this work.

## Author contributions

**Conceptualization:** Ryan Ka Hei Hung, Patrick SH Yung, Dino Samartzis, Cliffton Chan, Claire Hiller, Veronika Schoeb, Brenton Surgenor, Arnold YL Wong.

**Data curation:** Samuel KK Ling, Esther Tsz Chun Cheung, Arnold YL Wong.

**Formal analysis:** Ryan Ka Hei Hung.

**Funding acquisition:** Cliffton Chan, Claire Hiller, Veronika Schoeb, Brenton Surgenor, Arnold YL Wong.

**Investigation:** Ryan Ka Hei Hung, Esther Tsz Chun Cheung, Arnold YL Wong.

**Methodology:** Ryan Ka Hei Hung, Dino Samartzis, Cliffton Chan, Esther Tsz Chun Cheung, Veronika Schoeb, Brenton Surgenor.

**Project administration:** Arnold YL Wong.

**Resources:** Claire Hiller, Arnold YL Wong.

**Software:** Veronika Schoeb.

**Supervision:** Patrick SH Yung, Samuel KK Ling, Dino Samartzis, Arnold YL Wong.

**Validation:** Samuel KK Ling, Dino Samartzis, Claire Hiller, Esther Tsz Chun Cheung, Veronika Schoeb, Brenton Surgenor, Arnold YL Wong.

**Visualization:** Brenton Surgenor, Arnold YL Wong.

**Writing – original draft:** Ryan Ka Hei Hung, Patrick SH Yung, Cliffton Chan.

**Writing – review & editing:** Ryan Ka Hei Hung, Patrick SH Yung, Samuel KK Ling, Dino Samartzis, Cliffton Chan, Claire Hiller, Esther Tsz Chun Cheung, Veronika Schoeb, Brenton Surgenor, Arnold YL Wong.

## Supplementary Material
