# Supplementary material for: Prevalence of dance-related injuries and associated risk factors among children and young Chinese dance practitioners
Source: Medicine (Baltimore). 2023 Nov 24;102(47):e36052. doi: 10.1097/MD.0000000000036052 (PMC10681500; doi:10.1097/MD.0000000000036052)
Supplement: Supplementary file 1 [file medi-102-e36052-s001.docx]

**Supplementary File: Questionnaire**

**Dance-related Injury Survey**

You are cordially invited to participate in this online survey conducted by a group of researchers from The Hong Kong Polytechnic University, The Hong Kong Academy for Performing Arts, The Chinese University of Hong Kong, The University of Hong Kong and the University of Sydney, RUSH University, and Hong Kong Association for Dance Medicine & Science (This research project has been approved by the Human Subject Ethics Subcommittee at The Hong Kong Polytechnic University, approval number: HSEARxxxx). 

This questionnaire aims to collect data about how often dancers experience **dance-related injuries,** and to identify potential **risk factors that may be related to dance injury in Hong Kong**. Your participation can help dance schools, dance instructors and/or show directors to prepare better strategies to prevent dance injuries among amateur and professional dancers.

This questionnaire will **only need 12 to 20 minutes** to complete. It consists of **a maximum of 69 questions (the total number of questions depends on your answers)** related to your dance injury experience at different time periods, the cause of your major dance injury, your dance habits, your feeling towards the injury, and your dance experience. **Please attempt to complete the survey**and **click on the submit button (or return the paper-based questionnaire) at the end**. By clicking the submit button/returning the questionnaire, you agree to participate in this survey. 

Your answers will be kept strictly **confidential**. You are welcome to give positive and negative feedbacks at the end of this questionnaire.
We are grateful for your participation.

**Demographic information**

1. What is your age?

Ans:

- Don’t want to disclose

2. What is your gender?

- male

- female

- Don’t want to disclose

- others: _____________

3. If you are female, what age did your period start?

Ans:

- Not yet

- Don’t want to disclose

- Not applicable because I am a male

4. What is your height in meters?

Ans:

- Don’t want to disclose

5. What is your weight in kilograms?

Ans:

- Don’t want to disclose

6. What is your highest education level?

- Primary

- Secondary

- Tertiary

- Tertiary (Specialized in dancing e.g. Hong Kong Academy for Performing Arts)

- Professional degree (PCLL, MBBS, etc)

- Masters level

- PhD level

- Others:

- Don’t want to disclose

7. What is your ethnicity?

- Chinese/Taiwanese/Hongkongers

- Caribbean

- Southeast Asian

- Indian

- Pakistanian

- Japanese

- Korean

- Caucasian

- African

- Hispanic

- Others: ______________

- Don’t want to disclose

8. Does you father dance?

- Yes, he is a professional dancer

- Yes, he is an amateur dancer

- No, he has stopped dancing

- No, he has stopped dancing because of a dance injury

- No, he has never danced

9. Has your father suffered from dance injuries?

- Always

- Often

- Sometimes

- Seldom

- Never

10. Does you mother dance?

- Yes, she is a professional dancer

- Yes, she is an amateur dancer

- No, she has stopped dancing

- No, she has stopped dancing because of a dance injury

- No, she has never danced

11. Has your mother suffered from dance injuries?

- Always

- Often

- Sometimes

- Seldom

- Never

12. What is your occupation?

- Student

- Office worker

- Full-time dancer (18 hours or more per week)

- Part-time dancer (less than 18 hours per week)

- Professional (e.g. accountant, lawyer)

- Healthcare professional (e.g. physician, physiotherapist)

- Manual worker

- A job that involves a lot of standing or walking (e.g. salesperson)

- Housewife

- Others: _______________

- Don’t want to disclose

13. What was your average monthly income in the last 12 months (for professional dancers or dance instructors only)?

- Answer:

14. How would you describe your physical activity level in the last 7 days?

- Sedentary (little or no exercise, no moderate- or vigorous intensity physical activity)

- Insufficient activity (Some activity but insufficient to reach the level of moderate or vigorous level)

- Moderately active (> 150 mins of aerobic moderate-intensity physical activity (e.g. brisk walking, yoga, swimming, cycling) OR > 75 minutes of aerobic vigorous-intensity physical activity (e.g. jogging, aerobic dance, fast swimming or cycling) OR equivalent combinations of moderate and vigorous activity, aerobic activity should be in bouts of > 10 minutes)

- Very active (> 300 minutes of moderate aerobic physical activity OR > 150 minutes of vigorous aerobic physical activity, OR equivalent combinations of moderate and vigorous-intensity activities)

- Extremely active (Exceeding the very active physical activity level (e.g. professional athlete)

- Don’t want to disclose

15. Do you have any health issue/s that requires regular medical follow-up (e.g., asthma, hypertension, muscle or joint pain)? If yes, please specify:

- Yes, Ans: _______________________________

- No

- Don’t want to disclose

16. Do you have medical insurance?

- Yes

- No

17. Have you been diagnosed with scoliosis (a C- or S-shaped curve of the spine)?

- Yes

-No (Question 67)

- Don’t want to disclose

18. At what age were you diagnosed with scoliosis?

- Ans: _______________________________

- Don’t want to disclose

19. Do you wear a brace?

- Yes

- No (Question 67)

20. If you wear a brace, how many hours per day?

- ____________hours

21. How many months have you worn a brace?

- _____________ months

22. Have you been diagnosed with other muscle or joint probems that may affect your dancing?

- Yes: _______________________

-No

- Don’t want to disclose

**Dance history**

23. Which dance genres do you practice? (You can select one or more)

- Ballet

- Jazz

- Contemporary dance

- Hiphop

- Urban dance

- Chinese dance

- Latin

- Ballroom dance

- Belly dance

- Breakdance

- Others: ___________

- Don’t want to disclose

24. What proportion of time do you spend doing each of the following forms of dance? (Please ensure the total number adds up to 100%)’.

- % Ballet

- % Jazz

- % Contemporary dance

- % Hiphop

- % Urban dance

- % Chinese dance

- % Latin

- % Ballroom dance

- % Belly dance

- % Breakdance

- % Others: ___________

- Don’t want to disclose

25. What is your dance experience? (You can choose more than 1 answer)

Professional = a person who is trained and skilled with qualification of at least one form of dance or employed in a professional capacity to perform in a variety of dance settings

Pre-professional = a person who is undergoing training to become a professional dancer

Amateur = recreational dancers [the level based on your highest success (examination results/competition results) or the recognition among your peers]

- Full-time professional (18 hours or more per week)

- Part-time professional (less than 18 hours per week)

- Pre-professional

- Full-time dance instructor (18 hours or more per week)

- Part-time dance instructor (less than 18 hours per week)

- Amateur (Advanced level)

- Amateur (Intermediate level)

- Amateur (Beginner level)

26. In the last 12 months, what were your average hours of dance practice in a normal week (before your injury/s if any)?

Ans:

- Never (Go to Question 28)

- Don’t want to disclose

27. How many percentage of your time spend doing each of the following dance practice? (Please ensure the total number adds up to 100%)’.

- % dance classes/practicing dance skills

- % rehearsal

- others:

28. How many years have you undertaken regular dance practice (excluding those years without regular practice)?

Ans:

- Don’t want to disclose

29. In the last 12 months, how many dance performances did you undertake?

- Ans:

- Never (go to Question 32)

- Don’t want to disclose

30. If you summarize all the hours in performance over the last 12 months, how many hours did you do? (including multiple pieces of dance)

Ans:

- Don’t want to disclose

- Not applicable

31. How many years have you been performing regularly?

Ans:

- Never

- Others: ________

- Don’t want to disclose

32. In the last 12 months, how many dance competitions did you participate in?

- Ans:

- Never

- Don’t want to disclose

33. How many times have you participated in dance competitions since you started dance practice?

- Ans:

- Never

- Don’t want to disclose

34. Are you a dance instructor/teacher?

- Yes

- No (Go to Question 38)

35. Which main dance genre do you teach?

- Ballet

- Jazz

- Contemporary dance

- Hiphop

- Chinese dance

- Latin

- Ballroom dance

- Belly dance

- Breakdance

- Others:___________

- Don’t want to disclose

36. Which other dance genre(s) do you teach (for dance instructors only)? (You can choose more than 1 answer)

- Ballet

- Jazz

- Contemporary dance

- Hiphop

- Chinese dance

- Latin

- Ballroom dance

- Belly dance

- Others:

- Breakdance

- Others:___________

- Don’t want to disclose

37. In the last 12 months, how many hours of dance do you teach in a normal week (for dance instructors only)?

Ans:

- Don’t want to disclose

- Not applicable

**Dance-Related injury**

Please read the following before answering the following questions:

A dance-related injury in this survey is defined as any physical (e.g. numbness, weakness, pain, tingling or other physical symptoms) or psychological condition (e.g. fear, distress) following an injury that prohibits a person from fully participating in scheduled dance activities (practice or performance) that would have happened otherwise.

**Dance-related injuries in the last 12 months**

38. In the last 12 months, did you experience dance injuries?

- Yes

- No (Go to Question 59)

- Don’t want to disclose

39. How many times have you suffered from dance injuries in the last 12 months?

- New injuries: ________ times

- Re-injury: _________times

- Don’t want to disclose

40. What was your most significant dance injury in the last 12 months, and describe how this affects your dance practice and/or teaching?

Ans:

- Don’t want to disclose

41. When did you sustain the dance injury that you mentioned in Q40 (you can choose more than 1 answer)?

- Practice (technique classes, self-practice)

- Rehearsal

- Performance

- Competitions

- Not sure

- Others: _______

42. Please rate the average pain severity of the most significant dance injury mentioned in Q40.

|  |  |  |  |  |  |  |  |  |  |  |  |  |  |  |  |  |  |  |  |
| --- | --- | --- | --- | --- | --- | --- | --- | --- | --- | --- | --- | --- | --- | --- | --- | --- | --- | --- | --- |

0 1 2 3 4 5 6 7 8 9 10

No pain Worst imaginable

43. Did you have a previous history of dance injury as you mentioned in Q40?

- Yes

- No (Go to Question 45)

44. How many times did you have the same injury as you mentioned in Q40?

- Ans: _______

45. How many days did you skip full dance practice following this significant injury?

Ans:

- Don’t want to disclose

46. How many days could you not participate in full dance performance/competition following that most significant injury?

Ans:

- Don’t want to disclose

47. Apart from the most significant injury mentioned previously, please identify all body parts that has been injured because of dancing in the last 12 months? (You can select more than one answer; If you have not suffered from an injury in that region, leave the scale BLANK and move onto the next region)

| Body region | | Percent recovery from a previous injury (0 = not recovered at all; 100 = fully recovered) |
| --- | --- | --- |
| Head | |  |
| Neck | |  |
| Mid back | |  |
| Lower back | |  |
| Abdomen | |  |
| Shoulder/clavicle | Left |  |
|  | Right |  |
| Upper arm | Left |  |
|  | Right |  |
| Elbow | Left |  |
|  | Right |  |
| Forearm | Left |  |
|  | Right |  |
| Wrist/fingers | Left |  |
|  | Right |  |
| Upper arm | Left |  |
|  | Right |  |
| Chest/ribs | Left |  |
|  | Right |  |
| Pelvis/buttock | Left |  |
|  | Right |  |
| Thigh | Left |  |
|  | Right |  |
| Knee | Left |  |
|  | Right |  |
| Lower leg | Left |  |
|  | Right |  |
| Ankle | Left |  |
|  | Right |  |
| Foot/toes | Left |  |
|  | Right |  |
| Others |  |  |

48. If you suffered from injury in the above body region(s), please give details relating to these injuries (e.g. types of injuries such as concussion, sprain, overuse, etc.).

- Ans: _____________

49 How did you manage your dance injuries in the last 12 months? (You can select more than 1 answer)

| Strategy | Effectiveness with injury management (0 = no effect at all; 100 = greatest effect of all) |
| --- | --- |
| Ignore it and continue to dance |  |
| Rest |  |
| Ice, compression and elevation |  |
| Heat |  |
| Take over the counter painkillers |  |
| Take painkiller from doctor |  |
| Plaster |  |
| Splint/braces |  |
| Stretch |  |
| Exercise |  |
| Others: |  |

50. If you sought advice/consultation from others to manage your injury, please indicate the professional you saw and the effectiveness of this in the last 12 months? (You can choose more than 1 answer)

| People | Consulted | Effectiveness with injury management (0 = no effect at all; 100 = greatest effect of all) |
| --- | --- | --- |
| Don’t want to disclose |  | |
| I did not seek advice from others |  |  |
| Dance instructor | Yes / No |  |
| General practitioner | Yes / No |  |
| Friends | Yes / No |  |
| Bonesetter | Yes / No |  |
| Massage therapist | Yes / No |  |
| Physiotherapist | Yes / No |  |
| Psychologist | Yes/ No |  |
| Orthopedic surgeon | Yes / No |  |
| Rehabilitation doctor | Yes / No |  |
| Chiropractor | Yes / No |  |
| Osteopath | Yes / No |  |
| Pharmacist | Yes / No |  |
| Traditional Chinese Medicine practitioner | Yes / No |  |
| Others |  |  |

51. How long after you injured yourself did go to see the first medical professional (days)?

Ans: _________

Not applicable

52. Why didn’t you see the first medical professional right away?

- I thought the injury was not that serious until it did not recover by itself

- I did not trust medical professionals

- I did not want to wait for a long line up

- I did not have a health insurance

- My insurance did not cover such medical expenses

- Don’t want to disclose

- Not applicable

- Others:

53. How many days did you miss full dance practice/performance/competition because of dance injuries in the last 12 months?

Ans:

- Don’t want to disclose

- Not applicable

54. During the last 12 months, to what extent did your dance injury affect your daily activities (e.g. study, non-dance work, social activities)?

- Not limited at all

- Slightly limited

- Moderately limited

- Very limited

- Very disabling

55. What were the estimated medical expenses for all the dance injuries (HKD) in the last 12 months?

Ans: _______

- Zero

- Don’t want to disclose

- Not applicable because I did not sustain any injury

56. What was the estimated amount of income loss (HKD) due to dance-related injury work absenteeism (not limited to professional dancers) in the last 12 months?

Ans: ______

- Zero

- Don’t want to disclose

- Not applicable because I am not working

57. In the last 12 months, how many days or weeks were you unable to dance at your optimal level of performance due to injury?

- Ans:_______weeks_______days

- Don’t want to disclose

58. In the last 12 months, have you adopted any dance injury prevention techniques and how effective do you think these are techniques at preventing dance injuries? Even if you do not adopt them, please estimate the effectiveness of each strategy.

| Strategy | Which one of these strategies do you adopt to prevention dance injuries | Perceived effectiveness with injury prevention (0 = no effect at all; 100 = greatest effect of all) |
| --- | --- | --- |
| Don’t want to disclose |  |  |
| No strategy |  |  |
| Warm up |  |  |
| Cool down |  |  |
| Strengthening of big muscle groups |  |  |
| Taking regular short breaks during practice |  |  |
| Pilates |  |  |
| Nutrition and Dieting |  |  |
| Others: |  |  |
| Others: |  |  |

**Dance-Related injuries in the lifetime**

59. In your lifetime (excluding the last 12 months), have you sustained dance-related injuries (e.g. during training, competition, performance, etc.) that have interfered with dancing or participating in normal dance practice or performance in the following regions? (You can choose more than one answer)

- Never (Please go to Question 62)

| Body region | | Percent recovery from a previous injury (0 = not recovered at all; 100 = fully recovered) |
| --- | --- | --- |
| Head | |  |
| Neck | |  |
| Mid back | |  |
| Lower back | |  |
| Abdomen | |  |
| Shoulder/clavicle | Left |  |
|  | Right |  |
| Upper arm | Left |  |
|  | Right |  |
| Elbow | Left |  |
|  | Right |  |
| Forearm | Left |  |
|  | Right |  |
| Wrist/fingers | Left |  |
|  | Right |  |
| Upper arm | Left |  |
|  | Right |  |
| Chest/ribs | Left |  |
|  | Right |  |
| Pelvis/buttock | Left |  |
|  | Right |  |
| Thigh | Left |  |
|  | Right |  |
| Knee | Left |  |
|  | Right |  |
| Lower leg | Left |  |
|  | Right |  |
| Ankle | Left |  |
|  | Right |  |
| Foot/toes | Left |  |
|  | Right |  |
| Others |  |  |

60. In your lifetime, have you sustained any of the following dance injury/s that have interfered with your dancing or participation in normal dance practice or performance? (You can select more than one answer)

- Ligament Sprain

- Muscle Strain

- Fracture

- Dislocation

- Contusion bruise

- Overuse injury

- Concussion

- Abrasion/cut

- Blister

- Laceration/ Deep cut

- Fall injury

- Others: _____________

61. In your lifetime, who did you see to treat your dance injuries? (Please select three most common ones)

- No

- Bonesetter(s)

- Chiropractor(s)

- Dance instructor

- Friends in my dance organization(s)

- General practitioner(s)

- Massage therapist(s)

- Physiatrist(s)

- Pharmacists

- Physiotherapist(s)

- Psychologists

- Orthopedic surgeon(s)

- Osteopath(s)

- Traditional Chinese Medicine Doctor(s)

- Others: _______________

- Don’t want to disclose

- Not applicable because I did not sustain any dance injury so far

62. Following your dance injury, did you suffer from the following psychological/emotional problem? (You can choose more than 1 answer)

- Upset

- Fear

- Depression

- Anxiety

- Frustration

- Grumpiness

- Sleeplessness

- Distress

- Hopelessness

- Burnout

- Others: __________

- None of the above

- Don’t want to disclose

In the last two weeks, how often did you encountere the following problems?

63. Feeling nervious, anxious, or on edge

-Not at all

-Several days

-More than half the days

-Nearly every day

64. Not being able to stop or control worrying

-Not at all

-Several days

-More than half the days

-Nearly every day

65. Feeling down, depressed, and hopeless

-Not at all

-Several days

-More than half the days

-Nearly every day

66. Little interest or pleasure in doing thing

-Not at all

-Several days

-More than half the days

-Nearly every day

67. Do you have anything that you want to tell us about your dance injury or dance prevention?

- Ans:_____________

68. Optional: Please leave us your contact information if you want to receive a summary of results related to this research or to be contacted. Your contact information will be kept strictly confidential.

Yes, my email address: ____________ or phone number: __________

No

69. Would you like us to contact you for future dance related research?

Yes

No
